# Knowledge and practices: Risk perceptions of COVID-19 and satisfaction with preventive measures at workplace among maternity care providers in Pakistan

**DOI:** 10.18332/ejm/131864

**Published:** 2021-01-30

**Authors:** Rubina Izhar, Samia Husain, Muhammad A. Tahir, Sonia Husain

**Affiliations:** 1 Department of Gynaecology and Obstetrics, Aziz Medical Center, Karachi, Pakistan; 2 Karachi Medical and Dental College, Abbassi Shaheed Hospital, Karachi, Pakistan; 3 Aga Khan University Medical College, Karachi, Pakistan; 4 Aga Khan Hospital for Women, Karachi, Pakistan

**Keywords:** knowledge, personal protective equipment, practices, risk perception, job satisfaction, maternity care providers

## Abstract

**INTRODUCTION:**

While all healthcare services across the globe deferred non-urgent surgeries, labor wards provided maternity care during the COVID-19 pandemic continuously. This study assesses the knowledge and practices of obstetricians and midwives about personal protective equipment (PPE); their risk perception of COVID-19 and satisfaction with the preventive measures adopted at their workplace.

**METHODS:**

A questionnaire designed according to the World Health Organization’s advice on rational use of personal protective equipment for COVID-19 was administered to 452 Pakistani maternity care providers between 1 July and 30 July 2020.

**RESULTS:**

Most (85%) had adequate knowledge and 78.8% had good practices regarding PPE use. The perceived risk of contracting COVID-19 was lower than for influenza and tuberculosis. Perceived risk of contracting COVID-19 was highest for outpatient clinics. Fewer midwives compared to obstetricians (23.3% vs 32.9 %, p=0.001) were satisfied with the job security provided. Only 19.5% were satisfied with the social distancing measures at their setups. Less than one-third (31%) were satisfied with the PPE available to them.

**CONCLUSIONS:**

The participants had good knowledge and practices regarding PPE. The perceived risk of contracting COVID-19 was lower than for contracting influenza; however, they were concerned about contracting COVID-19 in outpatient clinics and emergency rooms. They had poor satisfaction with the measures adopted by hospital managements regarding job security and social distancing.

## INTRODUCTION

Coronavirus SARS-CoV-2 disease (COVID-19) is a rapidly evolving pandemic. Pakistan reported its first case on 26 February 2020. Pakistan has 341753 cases and 6943 deaths and at the time of writing (10 November 2020) is in the ‘clusters of cases’ stage of transmission^1^. According to WHO cluster of cases refers to experiencing cases, clustered in time, geographical location and/or by common exposures. Therefore, in order to contain spread it is of utmost importance to practice preventive measures and maintain social distancing in these times^2^.

The government implemented strict measures and they were successful in containing the spread in the country. Pakistan was thus able to fight COVID-19 better than other countries in the region^3^.

Maternity services and maternity care providers were uniquely affected by the pandemic. While all healthcare services across the globe deferred non-urgent surgeries^4^, labor wards continued to provide maternity care during the pandemic. Most of the laboring women are not affected, however there is a risk of contracting COVID-19 while providing high-quality care to these women^5^.

Healthcare workers (HCWs) are constantly exposed to COVID-19. In Italy, 10% of confirmed cases were HCWs, and 20% in Spain^6^. In the US, 3% of HCWs were affected and 55% reported that the exposure took place in a healthcare setting^7^. Infection in HCWs can lead to outbreaks in healthcare facilities, therefore, ensuring their protection is of utmost importance^8^.

Women exhale deeply and vomit in the second stage of labor putting all maternity care providers (MCPs) at risk of contracting COVID-19. The situation in countries where universal testing does not take place potentially exposes the whole team to the virus. While suggestions for full personal protective equipment (PPE) and N95 masks for the second stage of labor have been given, no clarification from the American College of Obstetricians and Gynecologists has been published to date^9^. Transmission of COVID-19 to fetus and whether labor is an aerosol generating procedure is still ambiguous.

COVID-19 spreads primarily through respiratory droplets, therefore washing hands and using appropriate PPE is essential for reducing transmission. Non-adherence or poor knowledge and practices regarding infection control and prevention can lead to outbreaks in healthcare facilities. HCWs have been advised to receive training and practice in the correct donning and doffing of PPE to optimize outcomes in healthcare settings^10^.

However, training needs are not uniform in all populations. A baseline study to assess the current level of knowledge and practices is essential to identify gaps in knowledge and practice areas that need most improvement. Furthermore, training programs are not the only facets that need attention. Many studies have assessed the anxiety and risk perception of women^11^ and healthcare workers in general^12^ but data on risk perception of maternity care providers (MCPs) remains sparse. Moreover, no study to date has assessed the satisfaction of MCPs regarding preventive measures adopted at their workplace during the pandemic. Additionally, no data on knowledge and practices regarding use of personal protective equipment in this population exists. We therefore conducted this study to assess the knowledge and practices of obstetricians and midwives about personal protective equipment, their risk perception of COVID-19 and satisfaction with the preventive measures adopted at their workplace.

## METHODS

### Sample size

We calculated the sample size using EpiCalc-2000. Our calculation was based on the following assumption that the proportion of good knowledge would be 50%, level of confidence 95% and precision 5%, resulting in a sample size of 384. The sample size was then increased by 10% to compensate for missing items. Our study included 452 people in total.

### Study tool

A self-administered questionnaire was designed for the study after reviewing World Health Organization’s advice on rational use of personal protective equipment for COVID-19 and considerations during severe shortage^13^. Two authors from the research team validated the content and ensured that all questions were relevant. We pretested the questionnaire on 34 people recruited through WhatsApp. These responses were excluded from the final analysis. No problems were encountered and the internal consistency of each section was as follows: knowledge 0.71, practices 0.828, and satisfaction 0.72.

The questionnaire had the following sections: I) Sociodemographics including age, work experience, maternity care provider, workplace, highest educational degree; and questions such as, ‘Have you received any formal PPE training?’, ‘Are you working >8 hours a day?’, and major source of information about COVID-19. II) Knowledge, comprising 13 questions that covered PPE use in general and for each setting. Questions were answered by ‘yes’, ‘no’, or ‘don't know’. Knowledge score was calculated for the 13 questions with correct answers given a score of 1 point each and wrong answers or ‘don't know’ scoring 0, with a maximum possible score of 13. Participants had adequate knowledge if they had a score ≥10. III) Practices, involving 13 questions that assessed practices regarding PPE use in different settings (9 items) and practices regarding preventive measures during COVID-19 pandemic (4 items). Response to each item was assessed on a 5-point Likert scale as follows: 1=rarely, 2=occasionally, 3=sometimes, 4=mostly, and 5=always. The practice score was calculated as follows: 13 items with a maximum possible score of 65. Respondents were classified as having good practices if they replied mostly or always to the question. IV) Two risk perception scales, developed after a literature review by the research team. We asked all participants to estimate their risk of contracting COVID-19 virus during their duty hours. We included tuberculosis, flu, HIV/AIDS, hepatitis B and C, accident at workplace and food poisoning in the section. Another risk perception scale was devised to assess the estimated risk from different activities during the duty hours. Responses were assessed on a 5-point scale: 1=very unlikely to 5=very likely. V) Satisfaction, involving 5 questions including three questions that assessed respondents’ satisfaction to the preventive measures adopted at their setup during COVID-19 pandemic; their satisfaction towards job security (one item) and screening measures taken at the setup (one item). Response to each item was assessed on a 5-point Likert scale as follows: 1=very unsatisfied, 2=unsatisfied, 3=neutral, 4=satisfied, and 5=very satisfied.

### Data collection process

Due to restrictions on close contact and gatherings, we collected data online. We circulated an online survey and used the snowball sampling strategy to recruit participants. A Google form was used to enhance accessibility of the survey. Participants were asked to share the link with their respective networks. There were only two inclusion criteria of the survey: 1) working in a labor setting in Pakistan, and 2) being employed. All participants gave informed consent. The participation was voluntary and no compensation was given to any respondent. The participants could only submit their responses if all questions were answered. Data reported in this study were collected between 1 July and 30 July 2020.We did not collect any identifying information to ensure participant anonymity of the survey. This study was reviewed and approved by the Institutional Ethics Committee of Aziz Medical Center( IEC/AZIZ/160/2020).

### Statistical analysis

Once all necessary data were obtained and checked for completeness, they were coded and analyzed using Statistical Package for Social Science (SPSS) software version 23(IBM Corp., Armonk, NY). Simple descriptive analyses with means and standard deviations (SD) were used for numerical data, and frequencies and percentages for qualitative data. T-test for two independent samples and chi-squared test were used to compare quantitative and qualitative variables, respectively. A value p≤0.05 was considered statistically significant.

## RESULTS

### Sample characteristics

A total of 452 respondents completed the survey. Among the respondents, 280 were obstetricians and 172 were midwives. About half were aged <30 years, had received some training on PPE and with duty >8 hours. Around 36.3% worked in tertiary care private hospitals and 35% worked in public tertiary care hospitals. Of the participants, 38.9% had a fellowship, 25% had a Master’s degree and 56% used the WHO site as a major source of information during COVID-19 pandemic (Table 1).

**Table 1:** Basic characteristics of the maternal providers population, Pakistan 2020 (N=452)^a^

*Characteristics*		*n (%)*
**Age** (years)	≤30	240 (53.1)
	30–40	100 (22.1)
	40–50	56 (12.4)
	≥50	56 (12.4)
**Have you received any formal PPE training?**	No	184 (40.7)
	Yes	268 (59.3)
**Are you working >8 hours a day?**	No	216 (47.8)
	Yes	236 (52.2)
**Work experience** (years)	<5	80 (17.7)
	>9	320 (70.8)
	5–9	52 (11.5)
**Maternity care provider**	Midwives	172 (40.4)
	Obstetricians	280 (59.6)
**Workplace**	Public sector tertiary	160 (35.4)
	Secondary care	64 (14.2)
	Private tertiary hospitals	164 (36.3)
	Trust hospitals	64 (14.2)
**Highest educational degree**	Bachelor’s	72 (15.9)
	Fellow	176 (38.9)
	Master’s	116 (25.7)
	Student	88 (19.5)
**Source of information on COVID-19 you use the most**	Government sites and media	60 (13.3)
	World Health Organization (WHO)	256 (56.6)
	Journals	12 (2.7)
	News media	44 (9.7)
	Social media	80 (17.7)
**Knowledge score,** mean ± SD, range	10.62±1.33	7.00–13.00
**Practice score,** mean ± SD, range	55.10±7.54	28.00– 65.00
**Knowledge type**	Adequate	384 (85.0)
	Inadequate	68 (15.0)
**Practice type**	Good	356 (78.8)
	Poor	96 (21.2)

a Values are number (percentage) or mean ± standard deviation. PPE: personal protective equipment.

### Knowledge score and association with predictors

The mean knowledge score was 10.62±1.33. Most (85%) participants had adequate knowledge of personal protective equipment and preventive measures. Total correct responses were 81.67%, midwives 80.3% and obstetricians 82.53%. Knowledge score was significantly associated with age of the respondent (p=0.001), work experience (p=0.001), qualification of healthcare worker (p=0.023), workplace of the respondent (p=0.001), and major source of information (p=0.001) (Table 2).

**Table 2:** Association of knowledge with characteristics of study population of maternity providers, Pakistan 2020

*Characteristics*	*Knowledge score*		
	*Mean±SD*	*Range*	*p^†^*
**Age** (years)			
≤30	11.21±1.33	9.00–13.00	
30–40	10.72±1.29	8.00–13.00	0.001**
40–50	10.64±1.41	9.00–13.00	
≥50	10.43±1.30	7.00–13.00	
**Have you received any PPE training?**			
No	10.65±1.44	7.00–13.00	0.666
Yes	10.60±1.26	8.00–13.00	
**Are you working >8 hours a day?**			
No	10.61±1.33	7.00–13.00	0.899
Yes	10.63±1.34	8.00–13.00	
**Work experience** (years)			
<5	10.90±1.31	9.00–13.00	0.001**
>9	10.45±1.35	7.00–13.00	
5–9	11.23±0.98	10.00–13.00	
**Maternity care providers**			
Midwives	10.44±1.34	7.00–13.00	0.026*
Obstetricians	10.73±1.32	8.00–13.00	
**Workplace**			
Public sector tertiary care	10.97±1.22	8.00–13.00	0.001**
Secondary care	10.31±1.27	7.00–12.00	
Private hospital	10.49±1.26	8.00–13.00	
Trust hospital	10.37±1.67	8.00–13.00	
**Highest educational degree**			
Bachelor’s	10.83±2.05	7.00–13.00	0.235
Fellow	10.68±1.21	8.00–13.00	
Master’s	10.52±1.01	9.00–12.00	
Student	10.45±1.20	8.00–13.00	
**Source of information on COVID-19 you use the most**			
Government sites and media	10.47±1.16	9.00–13.00	0.001**
World Health Organisation	10.83±1.25	7.00–13.00	
Journals	10.00±1.48	8.00–11.00	
News media	9.82±1.42	8.00–12.00	
Social media	10.60±1.47	8.00–13.00	

SD: standard deviation.

* p<0.05.

** p<0.01.

† Two independent sample t-test.

PPE: personal protective equipment.

Only 27% knew that the reuse of any item is not adequate without decontamination, which should be done by trained staff. Only half of the respondents knew that respirator/mask use for prolonged periods increases the risk of touching the mask and if such contact occurs hand hygiene becomes mandatory. Only 56.6% knew that disposable lab coats or impermeable plastic aprons should only be used for a small duration of contact with a patient (Supplementary file, Table S1).

### Practice score and association with predictors

The mean practice score was 55.1±7.5. Overall, 386 (78.8%) respondents had good practices regarding PPE use and preventive measures. During first screening where temperature is recorded and no direct contact is involved, 68.1% respondents always or mostly maintained 1 m distance, or admitted to using a mask and eye protection. During second screening, where an interview is done to inquire about symptoms and travel history, 69% respondents maintained physical distance of at least 1 m, wore a medical mask, gloves and performed hand hygiene. Midwives compared to obstetricians (69.8% vs 84.3%, p=0.001) did not always or mostly inspect each PPE item before use. Moreover, they did not mostly or always remove mask/respirator when it became difficult to breathe through, or remove other PPE (/gown /face shield /goggles) when they became wet, soiled or damaged compared to obstetricians (79.1% vs 95.1%, p=0.001). Midwives mostly or always did not follow the guidelines for safe removal of mask/respirator (76.7% vs 94.3%, p=0.001) (Table 3).

**Table 3:** Practices regarding masks and general preventive measures during the pandemic among maternity providers, Pakistan 2020 (N=452)

*Practices*	*Total*	*Midwives*	*Doctors*	*p^†^*
	*Good ^a^*	*Good*	*Good*	
	*n %*	*n %*	*n %*	
I inspect each item before use to be sure it is in good condition with no degradation, tears or wear that could affect performance.	356 (78.8)	120 (69.8)	236 (84.3)	0.001**
When providing direct care to COVID-19 patients, in the absence of aerosol generating procedures, I use a medical mask, gown, gloves, eye protection (goggles or face shield) and perform hand hygiene.	448 (99.1)	172 (100)	276 (98.6)	0.115
When providing direct care to COVID-19 patients in settings where aerosol generating procedures are frequently in place, I use a respirator, gown, gloves, eye protection (goggles or face shield), apron and perform hand hygiene.	372 (82.3)	144 (83.7)	228 (81.4)	0.535
In preliminary screening not involving direct contact I maintain 1 m distance, when physical distance is not feasible, I use a mask and eye protection.	380 (84.1)	136 (79.1)	244 (87.1)	0.023*
During physical examination of patients without symptoms suggestive of COVID-19, I use PPE according to standard precautions and risk assessment and perform hand hygiene.	400 (88.5)	152 (88.4)	248 (88.6)	0.949
During physical examination of a patient with symptoms suggestive of COVID-19, I use a medical mask, gown, gloves, eye protection (goggles or face shield) and perform hand hygiene.	384 (85.0)	144 (83.7)	240 (85.7)	0.565
During first screening (temperature measurement) not involving direct contact, I maintain 1 m distance, when physical distance is not feasible and yet no patient contact, I use a mask and eye protection.	308 (68.1)	116 (67.4)	192 (68.6)	0.802
During second screening (i.e. interviewing patients with fever for clinical symptoms suggestive of COVID-19 disease and travel history), I maintain physical distance of at least 1m, wear a medical mask, gloves and perform hand hygiene.	312 (69.0)	116 (67.4)	196 (70.0)	0.568
If mask/respirator becomes difficult to breathe through or all other PPE (mask/respirator /gown /face shield /goggles) becomes wet, soiled, damaged, or exposed to splash of chemicals, infectious substances, or body fluids, I remove it.	404 (89.4)	136 (79.1)	268 (95.7)	0.001**
If displaced from the face for any reason or if the front of the respirator/mask is touched to adjust it, I follow the safe procedure for removal and do not touch the front of the respirator/mask.	396 (87.6)	132 (76.7)	264 (94.3)	0.001**
I perform hand hygiene frequently, using an alcohol-based hand-rub if hands are not visibly dirty or soap and water.	420 (92.9)	156 (90.7)	264 (94.3)	0.149
I use respiratory hygiene, i.e. cover my nose and mouth with a bent elbow or paper tissue when coughing or sneezing, dispose of the tissue immediately after use, and perform hand hygiene.	404 (89.4)	152 (88.4)	252 (90.0)	0.585
I refrain from touching my mouth, nose and eyes.	380 (84.1)	144 (83.7)	236 (84.3)	0.873

a Good = always and mostly.

* p<0.05.

** p<0.01.

† Chi-squared test.

### Risk perception of contracting COVID-19

Our participants perceived the risk of contracting COVID-19 lower than of hepatitis B, C, flu, tuberculosis while they perceived the risk of getting AIDS lower than COVID-19 (Table 4). According to the respondents, risk of contracting COVID-19 was highest for outpatient clinics and emergency rooms. They considered cesarean higher risk than delivery and laparoscopy. While ward rounds were perceived to be of lowest risk.

**Table 4:** Risk perception of contracting COVID-19 among maternity providers, Pakistan 2020

*Contracting COVID-19 compared to other diseases*	*Score ^a^*	*Likely/very likely*
	*Mean (SD)*	*n %*
COVID-19	2.41 (1.0)	72 (15.9)
Food poisoning	3.01 (1.0)	132 (29.2)
Accident at workplace	2.65 (1.0)	88 (19.5)
Tuberculosis	3.00 (0.9)	140 (31.0)
Flu	3.51 (0.9)	252 (55.8)
HIV/AIDS	1.50 (0.7)	12 (2.7)
Hepatitis C	2.68 (1.6)	184 (40.7)
Hepatitis B	2.43 (1.2)	124 (27.4)
**Contracting COVID-19 during procedures**		
Caesarean section	3.59 (1.0)	284 (52.8)
Delivery	2.99 (1.0)	156 (34.5)
Laparoscopic management	3.00 (0.9)	164 (36.3)
Ward rounds	2.49 (0.9)	84 (18.6)
Emergency room	4.43 (0.8)	432 (95.5)
Outpatient department	4.64 (0.6)	432 (95.4)

a Score range: 1 = very unlikely to 5 = very likely.

### Satisfaction with the PPE and other preventive measures adopted

Of the participating MCPs only 132 (29.2%) were satisfied or very satisfied with the job security provided by their setup during pandemic. Fewer midwives compared to obstetricians were satisfied with the job security provided (23.3% vs 32.9 %, p=0.001). Only few MCPs were satisfied with the social distancing at outpatient clinics and emergency rooms of their setups (19.5%). Midwives were more satisfied than obstetricians with the measures implemented (23% vs 17.2%, p=0.001).

Less than one-third (31%) were satisfied or very satisfied with the PPE available to them whereas more than half (55.8%) were satisfied or very satisfied with the measures women adopted while visiting and during admission. More than two-thirds of the MCPs were satisfied or very satisfied with the screening, testing and isolation rooms of their setups (68.1%). Fewer midwives were unsatisfied or very unsatisfied with the screening testing and isolation available than obstetricians (9.3% vs 18.5%, p=0.001) (Table 5).

**Table 5:** Satisfaction with preventive measures ensured and personal protective equipment provided among maternity providers, Pakistan 2020

*Satisfaction*	*Total (N=452) n (%)*	*Midwives (N=172) n (%)*	*Obstetricians (N=280) n (%)*	*p^†^*
**Are you satisfied with the administration of your setup regarding job security in pandemic?**				
Very unsatisfied	20 (4.4)	12 (7.0)	8 (2.9)	0.001**
Unsatisfied	136 (30.1)	28 (16.3)	108 (38.6)	
Neutral	164 (36.3)	92 (53.5)	72 (25.7)	
Satisfied	84 (18.6)	28 (16.3)	56 (20.0)	
Very satisfied	48 (10.6)	12 (7.0)	36 (12.9)	
**Are you satisfied with the social distancing ensured at outpatient clinics and emergency room of your setup?**				
Very unsatisfied	44 (9.7)	4 (2.3)	40 (14.3)	0.001**
Unsatisfied	196 (43.4)	68 (39.5)	128 (45.7)	
Neutral	124 (27.4)	60 (34.9)	64 (22.9)	
Satisfied	52 (11.5)	28 (16.3)	24 (8.6)	
Very satisfied	36 (8.0)	12 (7.0)	24 (8.6)	
**Are you satisfied with the personal protective equipment available to you?**				
Very unsatisfied	0	0	0	0.164
Unsatisfied	164 (36.3)	64 (37.2)	100 (35.7)	
Neutral	148 (32.7)	60 (34.9)	88 (31.4)	
Satisfied	116 (25.7)	44 (25.6)	72 (25.7)	
Very satisfied	24 (5.3)	4 (2.3)	20 (7.1)	
**Are you satisfied with the measures women adopt while visiting and during admission?**				
Very unsatisfied	0	0	0	
Unsatisfied	84 (18.6)	40 (23.3)	44 (15.7)	0.067
Neutral	116 (25.7)	44 (25.6)	72 (25.7)	
Satisfied	188 (41.6)	60 (34.9)	128 (45.7)	
Very satisfied	64 (14.2)	28 (16.3)	36 (12.9)	
**Are you satisfied with the screening, testing and isolation rooms of your setup?**				
Very unsatisfied	20 (4.4)	0 (0)	20 (7.1)	0.001**
Unsatisfied	48 (10.6)	16 (9.3)	32 (11.4)	
Neutral	76 (16.8)	40 (23.3)	36 (12.9)	
Satisfied	160 (35.4)	52 (30.2)	108 (38.6)	
Very satisfied	148 (32.7)	64 (37.2)	84 (30.0)	

** p<0.01.

† Chi-squared test.

## DISCUSSION

### Main findings

The present study shows adequate knowledge of personal protective equipment and optimal practices in participating MCPs. Their perceived risk for contracting COVID-19 was lower than flu, tuberculosis, and hepatitis B and C. The perceived risk was greatest for outpatient clinics and emergency rooms. The satisfaction with PPE provided was low and the preventive measures adopted at workplace and the job security offered during the pandemic were unsatisfactory.

### Interpretations

COVID-19 has created an emotional and economic crisis worldwide. Amidst the chaos, healthcare workers continue to provide quality care. However, they are as vulnerable as others to the effects of the pandemic. Failure of proper PPE use can lead to hospital outbreaks^14^. One woman comes into contact with many HCWs and so the spread can be exponential in these circumstances. Therefore, knowledge of PPE in the maternity care sector is of absolute importance. Our participants had adequate knowledge, though reuse and decontamination were identified as gaps in the knowledge. Another area of concern was knowledge on use of disposable lab coats or impermeable plastic aprons. There is room for some improvement that could be addressed by designing training sessions catered to their specific needs. Just over one-third of healthcare workers across Australia^15^ and New Zealand^16^ indicated that their organization provided training in the use of high-level PPE. The majority of these respondents were from public hospitals. In our study, knowledge score of PPE use was higher in tertiary care public sector HCWs. In a recent study from Australia^17^, 70% of respondents had PPE training at new-staff orientation, 40% had received annual updates and 61% were provided training on request basis, at intervals ranging from monthly to every 5 years. In our study only 59.3% had received training on PPE use. Infection control and prevention programs rely heavily on training and monitoring competency of PPE use. An area for improvement was thus identified by our findings.

During the recent pandemic, HCWs have voiced their concerns for their safety and have shown lack of confidence in PPE use^18^. About 39% of training programs do not include a practice component due to the huge burden on resources that training requires^17^. In our study, most of the respondents had good practices though none of the 59.3% of respondents who received training had a hands-on component in the course.

CDC recommends placing a physical barrier made of glass or plastic to separate triage personnel and possible infectious patients to restrict close contact. Furthermore, examination rooms should be big enough to ensure social distancing^19^. During the first and second screening, our respondents neither maintained social distancing nor used appropriate PPE. This could be explained partially by the lack of proper social distancing measures ensured at their respective workplaces. Workplace sanitation and social distancing need to be ensured in addition to proper PPE^20^. Only one-third of the respondents were satisfied with the measures at their workplace. This shows that the management has failed to provide secure environment to these workers.

A study from Iran^21^ showed that PPE access is a predictor of physical health and job satisfaction. Only one-third of the respondents were satisfied with the PPE provided. In our study only two-thirds of the midwives inspected their PPE for damages before use. A logical explanation could be the availability and quality of products. If products are not available or are of poor quality, inspecting them before use is irrelevant. In a study from Hong Kong^22^ during the SARS pandemic, none of the staff that reported strict adherence to PPE use contracted the disease as opposed to those who missed at least one measure. This reaffirms the dictum that strict adherence is the key to containing spread and protecting frontline HCWs. Pakistan imported a lot of PPE from China and later started manufacturing most equipment locally. However, the cost of quality products remains high. Most of the institutes issue PPE for a fixed duration and replacement is not guaranteed until the designated time. In-depth interviews of the respondents should be conducted to answer these questions.

A study from China recently reported that under the risk of contact with suspected infected patients, HCWs show worse IPC behaviors. These behaviors may result from higher work load and insufficient supplies and resources among them^23^. More than half of our study respondents were working >8 hours per day. The midwives in our study were less likely to remove PPE if it became wet or soiled than the obstetricians. The use of facemasks and respirators is difficult and demands discipline. Photographs of healthcare providers with marks on their faces due to extended mask use have been circulating since the beginning of the pandemic. This shows that the workers are in distress and the management needs to increase the workforce or ensure an adequate break during the shifts. They were not satisfied with the job security provided to them. Our study proves that administration needs to provide some benefits and ensure that staff feel supported. Increasing the number of HCWs to reduce workload has been suggested as an effective measure^23^. Workplace satisfaction is essential during these hard times. It is of paramount importance that the workforce remains motivated to deliver quality care and that their ability is not compromised due to fatigue and ill feelings towards the management.

The risk perception of our respondents was low. They considered COVID-19 to be less contagious than flu, tuberculosis and food poisoning. Pakistan is a developing country where tuberculosis is still rampant and safe drinking water is not readily available^24^. Their risk perception should be understood in the context of this setting. However, the perceived risk of contracting COVID-19 was greatest for outpatient departments and emergency rooms. This could be explained by the lack of proper social distancing measures implemented. Our respondents rated their level of being infected by COVID-19 lowest in ward settings. Masking can be ensured and distancing is easier in wards. More than half of our study population was satisfied with the measures patients adopted during admission and more than two-thirds were satisfied with the screening, testing and isolation rooms of their respective setups. Everyone needs to play a part to ensure social distancing measures are implemented. All needs to be done to prevent an outbreak. Social distancing, wearing masks and a good administration are needed to prevent outbreaks and contain spread.

### Strengths and limitations

This is the first study that assessed the risk perception of maternity care providers of contracting COVID-19 and the satisfaction of these frontline workers during the pandemic. The study had an adequate sample size. We conducted the survey online to ensure representation from different cities of the country. Midwives are underrepresented in the hospital-based studies and their voice remains unheard if a hospital-based approach is used for such surveys in developing countries. We located midwives through their social media and nursing schools. We devised a satisfaction scale for workers in this pandemic that can be used to assess satisfaction in different populations. We did not use a validated measure because these unprecedented times call for different measures.

Online surveys have the inherent limitation of respondents giving socially acceptable answers and a face-to-face interview should be done in future to ensure reliability of responses. However, it is still a useful contribution to guide authorities on the issues being faced by maternity care providers in their respective setups. The study was in a low-middle-income country, therefore the results cannot be generalized to high-income countries.

### Policy and recommendations

The maternity care providers are an important human resource for any country and need to be supported during these unprecedented times. Policies regarding job security during the pandemic should be adopted. Moreover, the social distancing and wearing of masks should be followed so that the contagion does not spread. Awareness programs regarding masks are a need of the hour. Training programs for healthcare workers incorporating videos should be used to improve adherence to infection prevention and control.

## CONCLUSIONS

The study shows adequate knowledge and optimum practices in MCPs. The participants’ perceived risk of contracting COVID-19 was lower than the risk of contracting influenza; however, they were concerned about contracting it in outpatient areas and emergency rooms. They had poor satisfaction with the measures adopted by hospital management regarding job security and social distancing.
