# Supplementary material for: Knowledge and practices: Risk perceptions of COVID-19 and satisfaction with preventive measures at workplace among maternity care providers in Pakistan
Source: Eur J Midwifery. 2021 Jan 30;5:3. doi: 10.18332/ejm/131864 (PMC7847223; doi:10.18332/ejm/131864)
Supplement: Supplementary file 1 [file EJM-5-3-s1.pdf]

Supplementary file 1: Knowledge Of Personal Protective Equipment (PPE) Use During COVID-19:

KNOWLEDGE ITEMS:	Total correct	Healthcare worker		P value[a]†
		midwives	doctors	
		correct	correct	
	N %	N %	N %	
K1. The PPE can be used without removing for up to 6h, when caring for a cohort of COVID-19 patients	348 (77.0)	120 (69.8)	228 (81.4)	0.017*
K2. Respirator/mask use for prolonged periods may increase the chance of health care workers touching the respirator or having inadvertent under-respirator touches; if respirator/masks are touched/adjusted, hand hygiene must be performed immediately	228 (50.4)	84 (48.8)	144 (51.4)	0.10
K3. Use of the same PPE by a HCW between a patient with COVID-19 and a patient who does not have COVID-19 is not recommended owing to the risk of transmission to another patient who would be susceptible to COVID-19	384 (85.0)	152 (88.4)	232 (82.9)	0.064
K4. Face shield with proper design to cover the sides of the face and below the chin should be used and should be used only in the critical emergency situation of lack of medical masks	440 (97.3)	160 (93.0)	280 (100.0)	0.001**
K5. Face shields are protective against direct exposure of mouth, nose and eyes to droplets; however protection depends on the design and on the positioning of HCW in relation to the patient	448 (99.1)	168 (97.7)	280 (100.0)	0.021*
K6. The extended use of gowns may increase the risk of transmission of other pathogens between patients	408 (90.3)	144 (83.7)	264 (94.3)	0.001**
K7. Disposable lab coats or impermeable plastic aprons should only be used for brief contact with the patients; should not be used for prolonged contact or when performing aerosol-generating procedures and support treatments	256 (56.6)	88 (51.2)	168 (60.0)	0.078
K8. Goggles or safety glasses used by health workers may increase the discomfort and fatigue of health care workers and skin tissue damage may occur to face with prolonged goggle use	448 (99.1)	172 (100.0)	276 (98.6)	0.146
K9.Goggles should be removed if they are contaminated by splash of chemicals, infectious substances, or body fluids and if goggles obstruct health care worker safety or visibility of health care environment or become loose	448 (99.1)	172 (100.0)	276 (98.6)	0.303
K10. Face shield and goggles may be cleaned immediately after appropriate doffing and hand hygiene is performed OR placed in designated closed container for later cleaning and disinfection	424 (93.8)	164 (95.3)	260 (92.9)	0.286
K11. Use of PPE is the most visible control used to prevent the spread of infection, it is only one of the IPC measures and should not be relied on as a primary prevention strategy	400 (88.5)	156 (90.7)	244 (87.1)	0.25
K12. The reuse of any item without a reprocessing/ decontamination process is considered inadequate and unsafe and should be performed by trained staff in the sterile services department of a health care facility or at bigger scale under standardized conditions	124 (27.4)	44 (25.6)	80 (28.6)	0.516
K13. Methods for reprocessing masks or respirators are neither well established nor standardized, and therefore should be considered only when there is critical PPE shortage or lack of PPE.	444 (98.2)	172 (100.0)	272 (97.1)	0.027*

a*p $<$ 0.05; **p $<$ 0.01 †chi square test

© 2021 Izhar R. et al
